# 746 Burn Provider Perceptions Before and After Implementation of a Pain and Sedation Clinical Practice Guideline

**DOI:** 10.1093/jbcr/irad045.221

**Published:** 2023-05-15

**Authors:** Vince Dryer, Kathryn Smith, Rosemary Paine, Raechell Kearney, Carolyne Falank, Damien Carter, Alexandra Billert, Lisa Murray

**Affiliations:** Maine Medical Center, Portland, Maine; Maine Medical Center, Scarborough, Maine; Maine Medical Center, Portland, Maine; Maine Medical Center, Portland, Maine; Maine Medical Center, Portland, Maine; Maine Medical Center, Portland, Maine; Maine Medical Center, Falmouth, Maine; Maine Medical Center, Portland, Maine; Maine Medical Center, Portland, Maine; Maine Medical Center, Scarborough, Maine; Maine Medical Center, Portland, Maine; Maine Medical Center, Portland, Maine; Maine Medical Center, Portland, Maine; Maine Medical Center, Portland, Maine; Maine Medical Center, Falmouth, Maine; Maine Medical Center, Portland, Maine; Maine Medical Center, Portland, Maine; Maine Medical Center, Scarborough, Maine; Maine Medical Center, Portland, Maine; Maine Medical Center, Portland, Maine; Maine Medical Center, Portland, Maine; Maine Medical Center, Portland, Maine; Maine Medical Center, Falmouth, Maine; Maine Medical Center, Portland, Maine; Maine Medical Center, Portland, Maine; Maine Medical Center, Scarborough, Maine; Maine Medical Center, Portland, Maine; Maine Medical Center, Portland, Maine; Maine Medical Center, Portland, Maine; Maine Medical Center, Portland, Maine; Maine Medical Center, Falmouth, Maine; Maine Medical Center, Portland, Maine; Maine Medical Center, Portland, Maine; Maine Medical Center, Scarborough, Maine; Maine Medical Center, Portland, Maine; Maine Medical Center, Portland, Maine; Maine Medical Center, Portland, Maine; Maine Medical Center, Portland, Maine; Maine Medical Center, Falmouth, Maine; Maine Medical Center, Portland, Maine; Maine Medical Center, Portland, Maine; Maine Medical Center, Scarborough, Maine; Maine Medical Center, Portland, Maine; Maine Medical Center, Portland, Maine; Maine Medical Center, Portland, Maine; Maine Medical Center, Portland, Maine; Maine Medical Center, Falmouth, Maine; Maine Medical Center, Portland, Maine; Maine Medical Center, Portland, Maine; Maine Medical Center, Scarborough, Maine; Maine Medical Center, Portland, Maine; Maine Medical Center, Portland, Maine; Maine Medical Center, Portland, Maine; Maine Medical Center, Portland, Maine; Maine Medical Center, Falmouth, Maine; Maine Medical Center, Portland, Maine; Maine Medical Center, Portland, Maine; Maine Medical Center, Scarborough, Maine; Maine Medical Center, Portland, Maine; Maine Medical Center, Portland, Maine; Maine Medical Center, Portland, Maine; Maine Medical Center, Portland, Maine; Maine Medical Center, Falmouth, Maine; Maine Medical Center, Portland, Maine

## Abstract

**Introduction:**

Pain and sedation management is a crucial yet challenging aspect of burn care. We sought to survey a multidisciplinary group of nurses (RN), physicians (MD), advanced practice providers (APP), and therapists (PT/OT) to determine perceptions of pharmacologic pain and sedation management before and after the implementation of a standardized clinical practice guideline (CPG) at our institution.

**Methods:**

A 28-question survey assessing knowledge, pain and sedation practices, and therapy participation was distributed to all intensive care unit (ICU) and non-ICU burn care providers at our institution before (n=135, 2019) and after (n=185, 2022) CPG implementation. Comparison of proportions was calculated using Chi-squared test.

**Results:**

Survey response was 61 (45%) before and 51 (28%) after CPG, with 18 participating in both. Most respondents (63%) had 0-5 years of experience caring for burn patients. Patients were perceived to have significant background pain >50% of the time by 69% of RN before vs 55% after (p=0.26). Intermittent opioid boluses were preferred to continuous infusions in intubated patients before (57%) and after (59%) CPG implementation (p=0.87). Perceived utilization of multimodal pain management was high (82%) amongst all responders, except for ICU RN (53%) (p=0.05). While < 30% of all providers believed analgesics were underutilized for procedural pain, non-ICU RN consistently reported patients were in significant pain 50-75% of the time during procedures (78% before vs 85% after, p=0.61). Perceptions of sedative underutilization were unchanged before and after CPG (ICU RN 58% vs 33%, p=0.15; non-ICU RN 69% vs 71%, p=0.91; MD/APP 69% vs 71%, p=0.95). PT/OT reported pain as the most common reason for nonparticipation in therapy sessions with one-third reporting underutilization of analgesics in both surveys. In the after survey, 50% agree we manage pain effectively. Areas for improvement included open communication between disciplines regarding pain and sedation plan, increased use of intermittent sedatives compared to continuous, and less IV opioid utilization with more focus on nonopioid therapy. Fifty percent of after survey respondents were aware of the CPG.

**Conclusions:**

Implementation of a CPG did not change perceptions of pain and sedation at our institution. This may be due to limited education surrounding CPG and overall lack of utilization. Future efforts should focus on education and communication between disciplines.

**Applicability of Research to Practice:**

Variability in provider perceptions of pain and sedation management in patients with burn injury remain despite implementation of a CPG.

